# Three Exposure Metrics for Fine Particulate Matter Associated With Outpatient Visits for Acute Lower Respiratory Infection Among Children in Guangzhou, China

**DOI:** 10.3389/fpubh.2022.876496

**Published:** 2022-06-09

**Authors:** Danxia Xiao, Wenchun Guo, Debo Xu, Jiamin Chen, Zhenyu Liang, Xiao Zhang

**Affiliations:** ^1^Department of Pediatrics, Guangdong Second Provincial General Hospital, Guangzhou, China; ^2^Department of Pediatrics, The First Clinical of College, Guangdong Medical University, Zhanjiang, China

**Keywords:** PM 2.5, air pollution, acute lower respiratory infection, China, children

## Abstract

Ambient fine particulate matter (PM_2.5_) is associated with an elevated risk of acute lower respiratory infections (ALRI). However, this association has not been examined using alternative exposure metrics. We collected outpatient data of patients with ALRI aged <14 years from the administrative database of a large tertiary hospital in Guangzhou, China, from 2013 to 2019. Ambient PM_2.5_ was measured using three metrics: (a) daily mean, (b) daily excessive concentration hours (DECH), and (c) hourly peak. Generalized additive models were fitted to estimate the excess risk (ER) associated with PM_2.5_. A total of 105,639 ALRI (35,310 pneumonia and 68,218 bronchiolitis) outpatient visits were identified during the study period. An interquartile range increment in PM_2.5_ DECH was consistently associated with the highest ER of ALRI-related outpatient visits: 12.30% (95% confidence interval [CI]: 9.49–15.18%), compared with 11.20% (95% CI: 8.34–14.13%) for daily mean and 9.73% (95% CI: 6.97–12.55%) for hourly peak. The associations between the three metrics of PM_2.5_ and ALRI-related outpatient visits were stronger in the cold season than in the warm season. Future studies should consider PM_2.5_ DECH as an alternative method of exposure measurement, in addition to daily mean and hourly peak concentrations of PM_2.5_.

## Introduction

Lower respiratory infections, including pneumonia and bronchiolitis, are the leading causes of death in children under the age of 5 years (1–3). In China, an estimated 55.8 million (95% uncertainty interval [UI]: 48.17 to 55.51 million) cases of lower respiratory infections and 1,85,264.33 (95% UI: 157651.46 to 212877.21) deaths due to them were reported in 2019. These infections represented a major health burden and were the leading cause of mortality among children aged <5 years in China (4).

Ambient particulate matter, especially particulate matter with an aerodynamic diameter of <2.5 μm (PM_2.5_), is associated with an increased incidence, hospital admission, and mortality of acute lower respiratory infections (ALRI) (5–12). However, these studies used daily mean concentrations almost universally as proxy measurements for ambient PM_2.5_. Although a few alternative measurements of PM_2.5_ concentrations were proposed by researchers (13–17), none have been applied in empirical research to investigate the association between different metrics of PM_2.5_ and the risk of ALRI-related outpatient visits.

In this study, we collected outpatient data from 105,639 patients with ALRI aged 14 years in a large tertiary hospital in Guangzhou, China over a consecutive observational period of seven years. We measured ambient PM_2.5_ concentrations using three different metrics (daily mean, daily excessive concentration hours [DECH], and hourly peak), and further examined the associations between these three metrics of PM_2.5_ and the risk of ALRI-related outpatient visits.

## Methods

### Acute Lower Respiratory Infection Data

From February 2013 to December 2019, ALRI outpatient data for patients aged <14 years were obtained from Guangdong Second Provincial General Hospital, which is one of the largest tertiary hospitals in Guangzhou, China (18). This administrative database set up by the hospital regularly collects data including demographics, medical conditions, and diagnosis codes (19–21). The diagnoses were completed by attending physicians and further validated by trained medical coders. The causes of outpatient visits were defined using the International Classification of Diseases, Tenth Revision (ICD−10) as follows (20, 22): ALRI (J12–J18 and J20–J22), pneumonia (J12–J18), and bronchiolitis (J20–J21).

### Air Pollution and Meteorological Data

The daily concentrations of air pollutants, including PM_2.5_, PM_10_, nitrogen dioxide (NO_2_), sulfur dioxide (SO_2_), and ozone (O_3_), were retrieved from 11 air monitoring stations in Guangzhou during the study period. The mean concentration of the air pollutants collected by the 11 air monitoring stations was used as the daily concentration of air pollutants. We used a linear interpolation approach (the “na. approx” function in “zoo” package in R) to impute missing data (0.78% of the total observation days). Considering the potential impact of weather on ALRI, we obtained daily meteorological data (mean temperature and relative humidity [RH]) from the National Weather Data Sharing System (http://data.cma.cn/).

### Exposure Metric

We compared the associations between PM_2.5_ and daily ALRI-related hospital admissions using three different exposure metrics: PM_2.5_ DECH, hourly peak concentration, and daily mean concentration. The definitions of these three metrics are available elsewhere (13–16). Briefly, DECH was developed by Lin et al. (23) and defined as the daily total concentration hours above a specific concentration level. Based on the reference Air Quality Guidelines (daily mean of 15 μg/m^3^ for PM_2.5_) formulated by the World Health Organization (WHO) (24), we calculated the DECH using the following formula (16):


$$\begin{array}{l} \;DECH=\sum_{i=0}^{23}C_{i}\\ C_{i}=\{\;\begin{array}{c} C_{i}-15,\;C_{i}\geq 15\;\\ 0,\;C_{i}<15 \end{array} \end{array}$$

where i is the hourly time of 1 day, C_i_ is the concentration of PM_2.5_ at a given time point, and ΔC_i_ is the difference between C_i_ and the threshold concentration (15 μg/m^3^).

Another metric, PM_2.5_ hourly peak concentration, was proposed to investigate the adverse effects caused by high levels of PM_2.5_. PM_2.5_ hourly peak concentration was defined as the maximum concentration of PM_2.5_ during a 24-h period on an observation day. The PM_2.5_ daily mean was the most commonly used definition of ambient PM_2.5_ concentration in the literature.

### Statistical Models

Following the design of previous time-series studies in the field of air pollution epidemiology research (10, 11, 25–27), the association between PM_2.5_ and ALRI-related hospital outpatient visits was estimated using generalized additive Poisson models. Public holidays, days of the week, and winter and summer vacations for students were controlled as categorical variables in the models. Temporal trends, temperature, and RH were adjusted for as smoothing splines. We also controlled for the number of doctors per day in the models. In line with prior studies (25–27), we chose six degrees of freedom (df) per year for temporal trends, six df for moving average temperature of the current day, and the previous 3 days (Temp03), and RH.

Considering the potentially delayed adverse effects of air pollution, different lag structures were assessed to examine potential lag effects. In the single-lag day models, we begin with the same day (lag0) up to a five-day lag (lag5) based on previous studies (11, 25). In the multi-day lag models, we considered the accumulated effects (moving averages for the current day and the previous one, two, and three days [lag01, lag02, and lag03]).

### Stratified Analyses

To investigate whether the health effects of PM_2.5_ on ALRI, differed by sex, age group (age <5 vs. 5–14 years), and season (warm vs. cold), we performed subgroup analyses stratified by these factors. The warm and cold seasons are defined as the periods from April to September, and from October to March, respectively. In subgroup analyses stratified by season, we chose 3 dfs per year for temporal trends as each season covers only half of the year. We tested whether the differences between strata were significant by calculating the 95% CI, according to previous studies (10, 11).

### Sensitivity Analyses

We conducted a set of sensitivity analyses to check the robustness of the main results to alternative modeling strategies for temporal and meteorological factors, as well as multiple two-pollutant models. To consider the potential collinearity caused by the inclusion of two highly correlated variables in the same model, we examined the correlation between independent variables. If the correlation was > 0.80, they were not included in the same two-pollutant models.

The main results were first estimated by altering the df for the temporal trends and meteorological variables (df alternating from five to eight). Second, gaseous air pollutants (SO_2_, NO_2_, and O_3_) were further adjusted in addition to PM air pollution using two-pollutant models (28, 29).

All statistical analyses and data visualization were conducted using R version 4.0.5. Statistical significance was set at *P* < 0.05.

## Results

### Characteristics of the ALRI Outpatient Visits, Air Pollutants, and Meteorological Variables

In 2,058 days of observations, from February 2013 to December 2019, we identified 105,639 outpatient visits of patients with ALRI and aged <14 years, among which 35,310 (33.4%) and 68,218 (64.6%) were due to pneumonia and bronchiolitis, respectively. Table 1 presents the descriptive statistics of the daily outpatient visits, concentrations of air pollutants, and meteorological variables analyzed. The mean daily number of ALRI outpatient visits during the study period was 38 (standard deviation [SD]: 18), among which 13 (SD: 10) were pneumonia-related visits and 25 (SD: 11) were bronchiolitis visits. The daily mean of PM_2.5_ during the study period was 35.3 μg/m^3^ (SD: 19.1), the mean of PM_2.5_ hourly peak concentrations was 49.8 μg/m^3^ (SD: 27.2), and the mean of PM_2.5_ DECH in 24 h was 443.0 μg/m^3^ (SD: 409.6).

**Table 1:** Summary statistics of acute lower respiratory infections outpatient visits, air pollutants, and meteorological variables.

	**Mean**	**SD**	**Percentile**				
			**Min**	**25th**	**50th**	**75th**	**Max**
**No. of daily outpatient visits**
ALRI	38	18	1	25	35	47	124
Pneumonia	13	10	0	7	11	17	73
Bronchiolitis	25	11	0	16	23	31	70
**Air pollution**, **μg/m**^**3**^
PM_2.5_ DECH	443.0	409.6	0.0	137.5	327.0	644.0	3143.0
PM_2.5_ hourly peak	49.8	27.2	7.0	30.0	43.0	63.0	236.0
PM_2.5_ daily mean	35.3	19.1	4.6	21.4	30.8	45.1	154.5
PM_10_	56.6	27.2	10.0	37.2	50.0	71.2	216.2
SO_2_	11.0	4.8	2.6	7.6	10.1	13.5	37.7
NO_2_	46.0	18.5	8.8	33.3	41.9	54.3	176.7
O_3_	49.6	27.8	3.5	27.9	45.9	66.4	189.0
**Meteorological variables**
Temperature, °C	22.3	5.8	1.8	18.2	24.0	27.2	30.7
Relative humidity, %	80.3	11.2	34.0	74.4	82.7	88.9	97.0

*ALRI, acute lower respiratory infections; SD, standard deviation; DECH, daily excessive concentration hours*.

**Figure 1:**
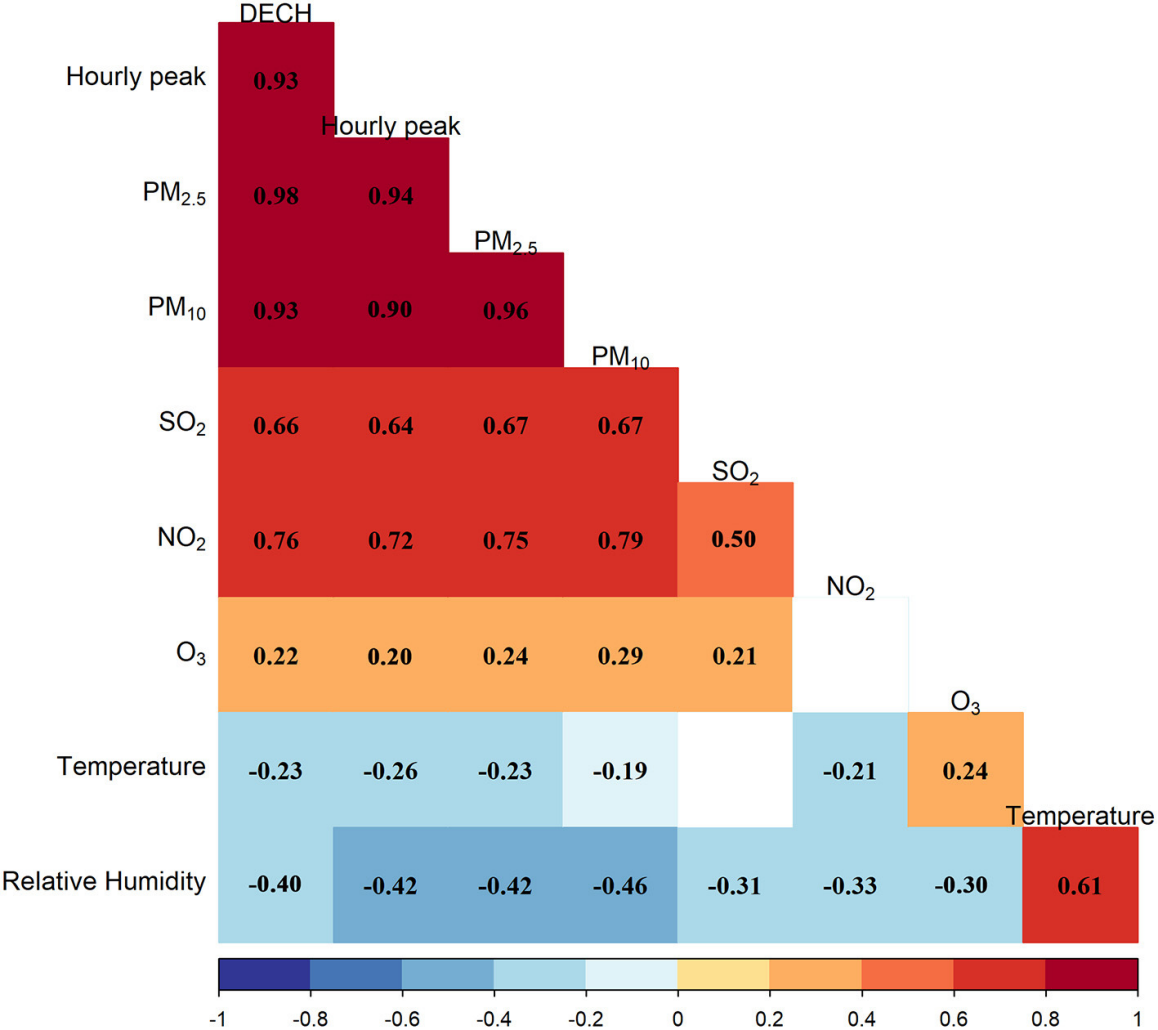
Pearson pairwise correlation plot of ambient air pollutants and meteorological factors.

Figure 1 shows the pairwise Pearson correlation coefficients for the air pollutants and meteorological factors. Ambient particulate matter air pollutants had Pearson correlation coefficients of over 0.9. The associations with SO_2_ and NO_2_ were moderately strong (Pearson correlation coefficients in the range of 0.6 and 0.8), while the absolute values of Pearson correlation coefficients for particulate matter and O_3_, as well as meteorological factors, were <0.5, indicating a lower strength of linear correlation.

### Associations Between the Three Metrics of PM and Risk of ALRI-Related Outpatient Visits

**Figure 2:**
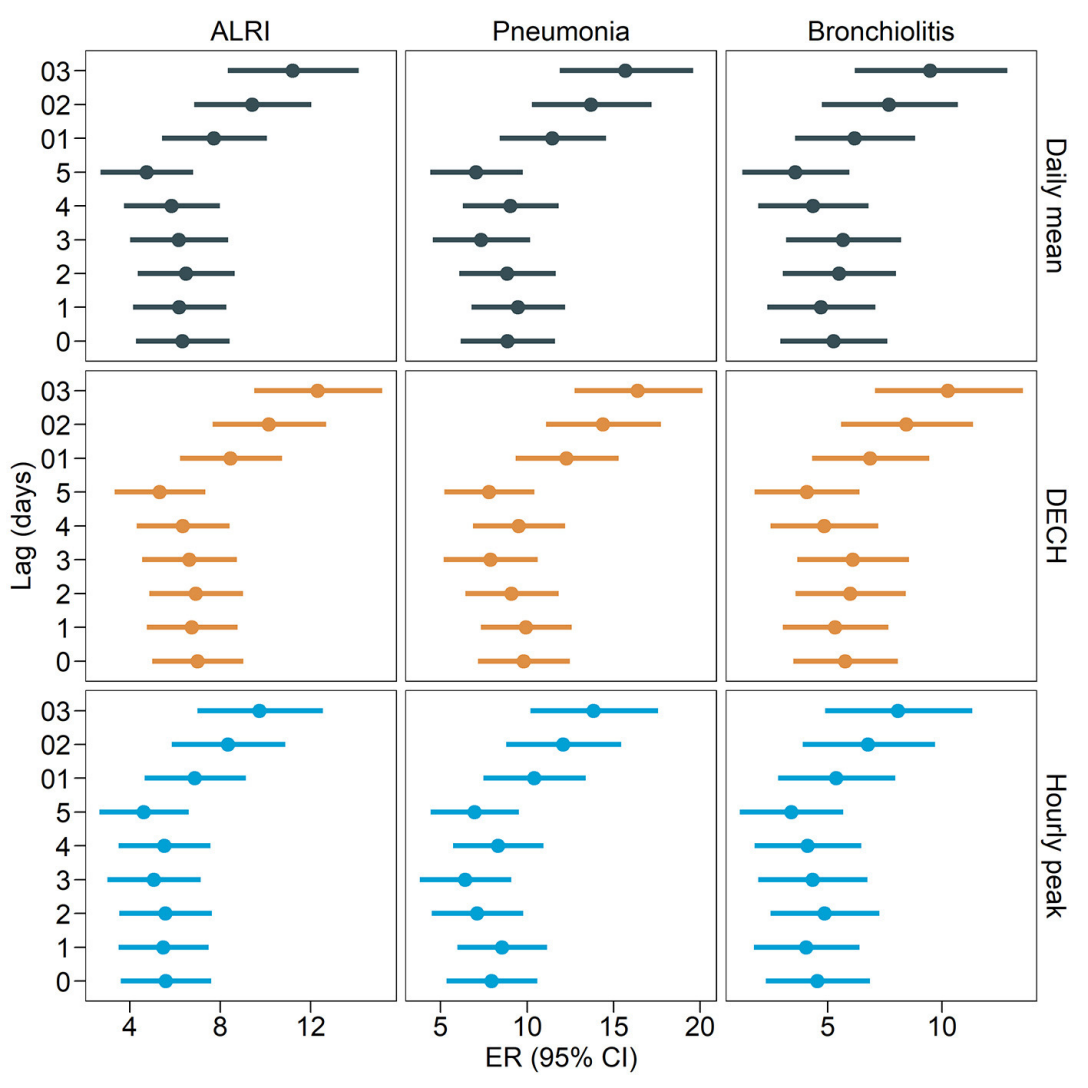
Excess risk (95% confidence intervals) of outpatient visits related to acute lower respiratory infections per interquartile range increment in ambient PM_2.5_ (daily mean, daily excessive concentration hours [DECH], and hourly peak) at different lag periods.

Table 2 and Figure 2 present the excessive risks (ER) and 95% CIs of ALRI, pneumonia, and bronchiolitis-related outpatient visits per interquartile range (IQR) increment in the three metrics of ambient PM_2.5_ exposure (daily mean, DECH, and hourly peak) at lag03. An IQR increment in PM_2.5_ DECH was consistently associated with the highest risk of outpatient visits for all three diseases [12.30% (95% CI: 9.49% to 15.18%) increase in ALRI, 16.32% (95% CI: 12.68% to 20.07%) increase in pneumonia, and 10.27% (95% CI: 7.09% to 13.55%) increase in bronchiolitis], followed by daily mean [11.20% (95% CI: 8.34% to 14.13%) increase in ALRI, 15.60% (95% CI: 11.81% to 19.52%) increase in pneumonia, and 9.50% (95% CI: 6.22% to 12.89%) increase in bronchiolitis] and hourly peak [9.73% (95% CI: 6.97% to 12.55%) increase in ALRI, 13.75% (95% CI: 10.11% to 17.50%) increase in pneumonia, and 8.09% (95% CI: 4.92% to 11.36%) increase in bronchiolitis].

**Table 2:** Excessive risk (95% confidence intervals) of outpatient visits of acute lower respiratory infection, pneumonia, and bronchiolitis per interquartile range increment in ambient PM_2.5_ (daily mean, daily excessive concentration hours [DECH], and hourly peak) at lag03 using single–pollutant and two–pollutant models.

**Pollutants**	**Models**	**Excessive risk (95% confidence intervals)**		
		**ALRI**	**Pneumonia**	**Bronchiolitis**
PM_2.5_ daily mean, interquartile range: 23.7 μg/m^3^				
	Single–pollutant model	11.20 (8.34, 14.13)	15.60 (11.81, 19.52)	9.50 (6.22, 12.89)
	Two–pollutant models			
	Control for SO_2_	9.33 (6.16, 12.59)	12.99 (8.81, 17.32)	7.84 (4.19, 11.62)
	Control for NO_2_	7.85 (4.50, 11.31)	12.10 (7.73, 16.65)	5.56 (1.77, 9.50)
	Control for O_3_	12.87 (9.80, 16.02)	16.96 (12.95, 21.11)	10.85 (7.38, 14.44)
PM_2.5_ DECH, interquartile range: 506.5 μg/m^3^				
	Single–pollutant model	12.30 (9.49, 15.18)	16.32 (12.68, 20.07)	10.27 (7.09, 13.55)
	Two–pollutant models			
	Control for SO_2_	10.47 (7.42, 13.62)	14.15 (10.14, 18.31)	8.95 (5.41, 12.61)
	Control for NO_2_	9.28 (6.05, 12.61)	13.48 (9.28, 17.84)	6.96 (3.30, 10.75)
	Control for O_3_	13.57 (10.63, 16.60)	17.55 (13.74, 21.50)	11.54 (8.20, 14.99)
PM_2.5_ hourly peak, interquartile range: 33 μg/m^3^				
	Single–pollutant model	9.73 (6.97, 12.55)	13.75 (10.11, 17.50)	8.09 (4.92, 11.36)
	Two–pollutant models			
	Con–rol for SO_2_	7.66 (4.64, 10.77)	10.91 (6.95, 15.02)	6.17 (2.68, 9.78)
	Control for NO_2_	6.02 (2.79, 9.36)	9.85 (5.67, 14.21)	3.77 (0.11, 7.56)
	Control for O_3_	11.13 (8.18, 14.15)	14.84 (11.02, 18.80)	9.18 (5.85, 12.61)

### Sensitivity Analyses

To test the robustness of our findings to alternative models and lag periods of exposure, we conducted two sensitivity analyses. We conducted a series of two-pollutant models by further including gaseous air pollutants (SO_2_, NO_2_, and O_3_), and the results (Table 2) were consistent with the main findings that PM_2.5_ DECH was associated with the highest risk of ALRI-related outpatient visits, followed by the daily mean, and hourly peak. To test the robustness of the results to different lag periods of PM_2.5_, we ran our main models for exposure measured at various lag periods (lag0 to lag5 and lag01 to lag 03). The ERs of ALRI-related outpatient visits were larger when PM_2.5_ was measured as moving averages (lag01, lag02, and lag03). The trend that PM_2.5_ DECH was associated with the highest risk of ALRI outpatient visits was generally consistent at different lag periods. To examine the consistency of the results for different dfs for the spline effects of temporal trends and temperature, we conducted the models using various dfs (Supplementary Table S1). The sensitivity analysis results showed that the estimates were larger at larger df, but the results were consistently larger for PM_2.5_ DECH than for daily mean and hourly peak, and all ERs remained statistically significant.

### Three Metrics of Ambient PM_2.5_ Associated With ALRI-Related Outpatient Visits in Subgroups

The ERs and 95% CIs of ALRI-related outpatient visits for the three metrics of PM_2.5_ by sex, age group, and season are shown in Table 3. We found that the associations between PM_2.5_ and ALRI-related outpatient visits were significantly larger in the cold season (October to March) than in the warm season (April to September), and this trend was consistent among the three metrics. The associations were insignificant during the warm season in several models. Although the ERs of ALRI outpatient visits were higher among men than among women and larger among children aged between five and 14 years than among those under 5 years, the differences across sex and age subgroups were not statistically significant.

**Table 3:** Excess risk and 95% confidence intervals of acute lower respiratory infection, pneumonia, and bronchiolitis for per interquartile range increment in ambient PM_2.5_ (daily mean, daily excessive concentration hours [DECH], and hourly peak) stratified by gender, age group, and season.

**Pollutants**	**Stratum**	**Excessive risk (95% confidence intervals)**		
		**ALRI**	**Pneumonia**	**Bronchiolitis**
PM_2.5_ daily mean
	Sex			
	Male (*N =* 47,966)	13.25 (10.01, 16.59)	18.58 (13.92, 23.43)	10.96 (7.37, 14.67)
	Female (*N =* 57,673)	8.27 (4.93, 11.71)	11.91 (7.17, 16.87)	7.18 (3.09, 11.43)
	Age			
	<5 (*N =* 41,799)	10.47 (7.53, 13.49)	14.39 (10.48, 18.45)	8.62 (5.27, 12.07)
	5–14 (*N =* 63,840)	13.11 (8.34, 18.09)	21.40 (12.60, 30.90)	13.34 (7.89, 19.07)
	Season			
	Warm (*N =* 40,922)	**1.45 (0.32, 2.60)**	**0.61 (−0.82, 2.05)**	2.23 (0.85, 3.62)
	Cold (*N =* 64,717)	**15.05 (10.87, 19.39)**	**24.17 (18.64, 29.97)**	11.28 (6.78, 15.95)
PM_2.5_ DECH
	Sex			
	Male (*N =* 47,966)	14.00 (10.88, 17.22)	19.02 (14.55, 23.66)	11.75 (8.28, 15.34)
	Female (*N =* 57,673)	9.69 (6.36, 13.12)	12.97 (8.40, 17.72)	7.90 (3.95, 12.00)
	Age			
	<5 (*N =* 41,799)	11.14 (8.30, 14.05)	15.04 (11.28, 18.93)	9.23 (5.99, 12.56)
	5–14 (*N =* 63,840)	16.97 (12.19, 21.96)	22.20 (13.80, 31.23)	14.61 (9.35, 20.12)
	Season			
	Warm (*N =* 40,922)	**1.65 (0.54, 2.76)**	**1.14 (−0.27, 2.56)**	**2.18 (0.84, 3.55)**
	Cold (*N =* 64,717)	**15.68 (11.69, 19.81)**	**24.34 (19.13, 29.79)**	**11.96 (7.65, 16.44)**
PM_2.5_ hourly peak
	Sex			
	Male (*N =* 47,966)	11.16 (8.05, 14.36)	16.12 (11.68, 20.73)	8.87 (5.42, 12.43)
	Female (*N =* 57,673)	8.27 (4.93, 11.71)	10.73 (6.18, 15.48)	6.91 (2.94, 11.03)
	Age			
	<5 (*N =* 41,799)	9.27 (6.43, 12.18)	12.96 (9.22, 16.84)	7.39 (4.15, 10.73)
	5–14 (*N =* 63,840)	13.11 (8.34, 18.09)	17.12 (8.68, 26.22)	11.22 (6.00, 16.70)
	Season			
	Warm (*N =* 40,922)	**0.30 (−0.68, 1.29)**	**−0.14 (-1.40, 1.15)**	**0.76 (−0.43, 1.97)**
	Cold (*N =* 64,717)	**15.02 (10.77, 19.43)**	**24.47 (18.81, 30.40)**	**11.07 (6.52, 15.81)**

*The bold type represents the statistically significant differences (p <0.05)*.

*Warm seasons, April to September; cold seasons, October to March*.

## Discussion

In this time-series analysis of daily ALRI-related outpatient visits aged <14 years in Guangzhou, China, we examined the associations of three metrics of ambient PM_2.5_ (daily mean, DECH, and hourly peak) with the risk of ALRI-related outpatient visits. All three metrics of PM_2.5_ were associated with significantly elevated risks of ALRI-, pneumonia-, and bronchiolitis-related outpatient visits. More importantly, PM_2.5_ DECH exhibited consistently larger ER in all models and subgroup analyses than the more commonly used daily mean and hourly peak metrics. The associations of PM_2.5_ with ALRI-related outpatient visits were significantly stronger in the cold season than in the warm season.

Although extensive research has been conducted on the association between ambient PM_2.5_ and the risk of ALRI-related outpatient visits (5–12), investigations are lacking regarding the metrics used (DECH or daily peak). To our knowledge, this is the first study to examine the associations between the three metrics of PM_2.5_ and ALRI-related outpatient visits. Our findings revealed that PM_2.5_ DECH, a metric of total excessive exposure to PM_2.5_ using the WHO guidelines, exhibited the largest risk of ALRI-related (pneumonia and bronchiolitis) outpatient visits. This trend is consistent with that in previous studies that provided evidence that PM_2.5_ DECH exhibited larger effect sizes than the daily mean (11, 16, 23). The larger effect size with PM_2.5_ DECH may be explained by more finessed concentrations of PM_2.5_ measured at different hours in a day; DECH is a summation of excessive PM_2.5_ at each hour, while the hourly peak is the highest hourly concentration in a day and the daily mean does not account for the reference guideline. In addition, previous studies suggested that PM_2.5_ DECH showed a better model fit performance, as measured using the Akaike information criterion (16). PM_2.5_ DECH is also more flexible in terms of the adaptation of the reference concentration and can be computed per guideline concentration in different countries and regions. This is exceptionally helpful in the context of various inconsistent guidelines recommended by different organizations, such as the WHO standard and the United States National Ambient Air Quality Standards (24). Our findings suggest that PM_2.5_ DECH may serve as an alternative measure of ambient PM_2.5_ concentration to the daily mean.

An interesting result of this study is that the associations between ambient PM_2.5_ and the risk of ALRI-related outpatient visits were significantly stronger in the cold season than in the warm season, which is in line with the findings of many previous epidemiological studies conducted in other regions and countries that reported a higher incidence of respiratory diseases attributable to air pollution (5, 11). One possible explanation is that lower temperatures in cold seasons may have synergistic effects on the adverse outcomes of ambient air pollution through mechanisms, such as impaired immunity of the local respiratory tract, poor air circulation, and slow air convection (12). The stronger associations in cold seasons may be partially explained by the influenza epidemic, which usually occurs during winter, but we were not able to control for this in our models owing to data unavailability.

Several biological mechanisms may explain the observed association between ambient particulate matter-related air pollution and elevated risk of ALRI-related outpatient visits in this study. Animal-based experimental studies have suggested that particulate matter induces inflammation in pulmonary cells through oxidant radical generation and further impairs lung function (30, 31). Together, these mechanisms indicate that exposure to ambient particulate matter may exacerbate lung function and prolong the recovery of lung cells from inflammation (5, 32), triggering an increased risk of ALRI within the short period observed in our study.

Previous environmental health studies predominately used daily mean concentration as the metric for ambient air pollution, given its simplicity in data collection and numeric calculation (12, 16, 25). The results of this study have the public health implication that DECH or daily peak can serve as alternative metrics of ambient air pollution and may exhibit larger effect sizes than daily mean concentrations. In addition, the epidemiological results, corroborated by several previous studies (5, 6, 10, 11), also suggest that more care should be provided to children during heavily polluted days to prevent ALRI-related outpatient visits and ameliorate the health outcomes.

This study has some limitations. First, the data used in this study were restricted to a single hospital in Guangdong, China. The limitations of the study sample may limit the generalizability of the findings to other populations. Second, this was a time-series study using the daily counts of ALRI as the outcome variable. The aggregated data make this an ecological study by design and are, therefore, subject to ecological fallacy. Third, because patient addresses were not available to the researchers, the exposure to PM_2.5_ was measured at the city level; ambient air pollution was not measured at an individual level, which may have led to exposure misclassification. Fourth, since we used the secondary administrative database designed by the hospital, a few important variables, such as indoor air pollution, smoking, economic status, and insurance, were missing from the analyses. Fifth, detailed daily data on influenza epidemics are not publicly available and cannot be statistically controlled for in our models; this may have led to residual confounding.

Nonetheless, this is the first study to investigate the association between three metrics of ambient PM_2.5_, and the risk of ALRI-related outpatient visits of patients aged <14 years. The results of ER and 95% CIs of the three metrics shed light on the adverse effects of ambient PM_2.5_, measured at different scales. Administrative outpatient data of 105,639 patients with ALRI spanning seven consecutive years were collected from a large tertiary hospital in Guangzhou, China, which resulted in relatively large sample size and statistical power.

## Conclusions

This time-series study found that PM_2.5_ DECH manifested larger effect sizes in the associations between ambient PM_2.5_ concentrations and ALRI-related outpatient visits of patients aged <14 years. Future studies may consider using PM_2.5_ DECH as an alternative method of exposure measurement, in addition to daily mean and hourly peak.

## Data Availability Statement

The data may be available upon requests to the corresponding author.

## Author Contributions

DX: conceptualization, investigation, visualization, writing—original draft, and writing—reviewing and editing. WG, DX, and JC: investigation, writing—reviewing and editing. ZL and XZ: investigation, visualization, and supervision. All authors contributed to the article and approved the submitted version.

## Conflict of Interest

The authors declare that the research was conducted in the absence of any commercial or financial relationships that could be construed as a potential conflict of interest.

## Publisher's Note

All claims expressed in this article are solely those of the authors and do not necessarily represent those of their affiliated organizations, or those of the publisher, the editors and the reviewers. Any product that may be evaluated in this article, or claim that may be made by its manufacturer, is not guaranteed or endorsed by the publisher.
