# Supplementary material for: Three Exposure Metrics for Fine Particulate Matter Associated With Outpatient Visits for Acute Lower Respiratory Infection Among Children in Guangzhou, China
Source: Front Public Health. 2022 Jun 9;10:876496. doi: 10.3389/fpubh.2022.876496 (PMC9218103; doi:10.3389/fpubh.2022.876496)
Supplement: Supplementary file 1 [file Table_1.DOCX]

**Table S1**. Associations between three metrics of ambient PM_2.5_ and risks of ALRI outpatient visits using different degrees of freedom for spline effects of temporal trends and temperature.

| Pollutants | Models | ALRI | Pneumonia | Bronchiolitis |
| --- | --- | --- | --- | --- |
| PM_2.5_ daily mean | |  |  |  |
|  | df=5 for temporal trends | 10.39 (7.37, 13.51) | 15.52 (11.58, 19.61) | 8.35 (5.00, 11.81) |
|  | df=7 for temporal trends | 11.44 (8.49, 14.49) | 15.21 (11.27, 19.30) | 9.91 (6.48, 13.45) |
|  | df=8 for temporal trends | 11.52 (8.67, 14.45) | 14.33 (10.49, 18.29) | 10.58 (7.24, 14.02) |
|  | df=5 for temperature | 11.19 (8.33, 14.11) | 15.60 (11.80, 19.52) | 9.46 (6.18, 12.83) |
|  | df=7 for temperature | 11.25 (8.34, 14.24) | 15.52 (11.72, 19.45) | 9.43 (6.12, 12.84) |
|  | df=8 for temperature | 11.52 (8.60, 14.54) | 15.69 (11.87, 19.64) | 9.67 (6.34, 13.10) |
| PM_2.5_ DECH | |  |  |  |
|  | df=5 for temporal trends | 11.25 (8.34, 14.24) | 16.23 (12.44, 20.15) | 9.42 (6.19, 12.75) |
|  | df=7 for temporal trends | 12.37 (9.53, 15.29) | 16.14 (12.35, 20.06) | 10.89 (7.56, 14.32) |
|  | df=8 for temporal trends | 12.76 (9.92, 15.67) | 15.30 (11.62, 19.09) | 11.28 (8.05, 14.60) |
|  | df=5 for temperature | 11.99 (9.23, 14.80) | 16.30 (12.67, 20.06) | 10.24 (7.07, 13.51) |
|  | df=7 for temperature | 12.07 (9.25, 14.94) | 16.25 (12.60, 20.02) | 10.22 (7.02, 13.52) |
|  | df=8 for temperature | 12.31 (9.48, 15.20) | 16.40 (12.74, 20.18) | 10.43 (7.21, 13.74) |
| PM_2.5_ hourly peak | |  |  |  |
|  | df=5 for temporal trends | 8.73 (5.82, 11.73) | 13.80 (10.01, 17.72) | 6.68 (3.45, 10.02) |
|  | df=7 for temporal trends | 9.83 (6.99, 12.75) | 13.00 (9.23, 16.91) | 8.48 (5.17, 11.89) |
|  | df=8 for temporal trends | 9.94 (7.18, 12.74) | 12.46 (8.76, 16.28) | 9.13 (5.89, 12.46) |
|  | df=5 for temperature | 9.73 (6.97, 12.54) | 13.77 (10.13, 17.52) | 8.05 (4.89, 11.31) |
|  | df=7 for temperature | 9.80 (7.01, 12.69) | 13.68 (10.04, 17.44) | 8.05 (4.85, 11.35) |
|  | df=8 for temperature | 10.02 (7.21, 12.90) | 13.68 (10.14, 17.55) | 8.25 (5.04, 11.55) |
